# Supplementary material for: Establishing a National Community of Practice for Newborn Screening Follow-Up
Source: Int J Neonatal Screen. 2021 Jul 26;7(3):49. doi: 10.3390/ijns7030049 (PMC8395728; doi:10.3390/ijns7030049)
Supplement: Supplementary file 1 [file IJNS-07-00049-s001.zip › IJNS-1273396-supplementary.pdf]

Supplementary File
IJNS- 1273396
Establishing a National Community of Practice for Newborn Screening Follow-Up

Table S1: National Webinars Hosted by the NewSTEPs Short Term Follow-up Workgroup

Date	Webinar Topic
January 2020	Establishing and reviewing cut-offs for analysis of new disorders
October 2018	Emergency Preparedness for Newborn Screening Programs
May 2018	Recap of In-Person Meeting
March 2018	Collaborating with Families & Providers in Community Birth Settings
January 2018	HIPAA Issues in Newborn Screening
November 2017	Family Experiences with Short Term Follow Up
September 2017	Spinal Muscular Atrophy
July 2017	The Power of Case Studies
May 2017	Long-term Follow-up Systems and Perspectives
March 2017	Update on CPT1a Deficiency in the Alaska Native Population
January 2017	Using Advisory Committees for Newborn Screening Programs
November 2016	Take-Aways from the Short Term Follow Up Stakeholders Meeting: False Negative, Co-location of Laboratory and Follow Up, Funding Challenges, Cystic Fibrosis Quality Improvement Meeting
September 2016	Newborn Screening Consultants/Linkage to Care
July 2016	Recap of the Cystic Fibrosis meeting/Timeliness in Newborn Screening Consideration for Cystic Fibrosis
May 2016	Follow Up for X linked Adrenoleukodystrophy.
March 2016	Using Infographics for Data and Parent Materials
January 2016	Hemoglobinopathy Follow-Up
November 2015	Collaborative Improvement and Innovation Network (CoIIN) for Timeliness in Newborn Screening
September 2015	Reducing Time from Referral to Treatment: Strengthening the Weak Links in the Newborn Screening Chain of Events/Illinois Newborn Screening Quality Improvement Initiatives/Virginia's Newborn Screening Transit Time Project`
July 2015	Newborn Screening for Congenital Adrenal Hyperplasia/Multistate Analysis of Single Tests or Routine Second Testing for CAH/Reducing False Positives and False Negatives with 2nd Tier Analysis.
May 2015	Improving Short- and Long-term Follow-up Efficiency through Implementation of an Internet Case Management System/Newborn Screening Short Term Follow-Up and the INSTEP Application
March 2015	The Controversy of Mild Congenital Hypothyroidism/Vermont Protocols for Follow-up of Mild Congenital Hypothyroidism/Feasibility of Providing Long Term Follow-Up for Congenital Hypothyroidism

Supplementary File
IJNS- 1273396
Establishing a National Community of Practice for Newborn Screening Follow-Up

January 2015	Short Term Follow-Up Theme/Non-SCID T Cell Lymphopenias During the First Year of NBS in Washington State/Diagnosis and Short Term Follow-Up of Cases found by TREC NBS/Winter Storm Knife
November 2014	Recap of Symposium Round Table Discussion
September 2014	Genetics Counselors Enhancing NBS Activities/The Role of a Clinical Genetic Counselor in NBS
July 2014	HIPAA Rule Change/Ethical Implications of Direct to Patient Test Reporting
May 2014	Electronic Vital Records and Screening System in SD/Genetic Services Short Term Follow-Up in NJ/Matching Vital Records and NBS Data in NYS/ Delaware Missed Babies
March 2014	Newborn Screening Education in Texas/A Parent's Perspective on Newborn Screening/Health Information Technology (HIT) Workgroup
January 2014	The Importance of Identifying False Negatives/Using a Secure Portal (SharePoint) for Communications and Data Collection
November 2013	Follow-up of Unsuitable and Borderline Specimens in NYS: When is Enough Enough?
